# Effect of Essential Oils from Ginger (*Zingiber officinale*) and Turmeric (*Curcuma longa*) Rhizomes on Some Inflammatory Biomarkers in Cadmium Induced Neurotoxicity in Rats

**DOI:** 10.1155/2018/4109491

**Published:** 2018-10-08

**Authors:** Ayodele Jacob Akinyemi, Philip Adeyemi Adeniyi

**Affiliations:** ^1^Department of Chemical Sciences, Biochemistry Unit, Afe Babalola University, Ado-Ekiti, Nigeria; ^2^Cell Biology and Neurotoxicity Unit, Department of Anatomy, College of Medicine and Health Sciences, Nigeria

## Abstract

Studies have revealed that anti-inflammatory agents could provide beneficial effect in lowering the incidence/progression of neurological diseases. Hence, this study sought to investigate the effect of essential oils from Nigeria ginger and turmeric rhizomes on some cytokines in cadmium induced neurotoxicity. The result revealed that essential oil from ginger and turmeric rhizomes exerts anti-inflammatory effect by preventing alterations of some cytokines/inflammatory biomarkers (IL-6, IL-10 and TNF-Alpha) levels and inhibits both hippocampus and prefrontal cortex acetylcholinesterase (AChE) and adenosine deaminase (ADA) activities (important enzymes relevant in the management/prevention of neurodegenerative diseases) in Cd treated rats. In conclusion, essential oil from ginger and turmeric rhizomes exerts anti-inflammatory properties in Cd induced neurotoxicity. The observed effect could be due to the volatile compounds as revealed by GC-MS analysis.

## 1. Introduction

Neuroinflammation simply referred to as inflammation of the nervous tissue is caused by a variety of assault to the brain ranging from infection, traumatic brain injury, toxic metabolites, or autoimmunity [1]. Recently, it has been reported to contribute to the pathogenesis of neurodegenerative diseases such as Alzheimer's disease, Parkinson's disease, and multiple sclerosis [1]. Neuroinflammatory processes have been linked to the activation of three common cytokines namely; interleukin-6 (IL-6), interleukin-1 beta (IL-1*β*), and tumor necrosis factor alpha (TNF-*α*) in the CNS [1]. Several heavy metals have been implicated in the role of neuroinflammation and Alzheimer's disease (AD) via formation of neurofibrillary tangles and amyloid-beta plaques [2, 3].

Cadmium (Cd) is well known for its neurotoxic potential and constitutes a serious global environmental health challenge [2]. Human is exposed to Cd via different sources such as primary metal industries, production of certain batteries, intake of contaminated food or water, and inhalation of tobacco smoke or polluted air [2]. Studies have shown that Cd can easily cross the blood brain barrier (BBB), a specialized structure composed of astrocytes and endothelial cells, inducing neuroinflammatory processes [2–4]. It has been reported by Ashok et al. [2] that Cd build up in the human body can induced neurotoxicity due to its high blood-brain barrier permeability. Neurological disorders such as memory impairments and hyperactivity in children may occur during Cd exposure [5]. Disturbances in neuroinflammatory processes have been noticed by Ashok et al. [2] after exposure to Cd. According to Wang and Du [6], the probable mechanism by which Cd induces neurological damage is via oxidative stress; however, the toxic effects of Cd are rather complex and still debated. Other mechanisms such as alterations in inflammatory processes and protein damage in neuronal cells have been proposed [5]. Hence, current research focuses on anti-inflammatory agents from either synthetic or food sources that can help alleviate heavy metal induced neurodegeneration.

Ginger otherwise known as the root of* Zingiber officinale *Roscoe is commonly used as spice and discovered to have diverse pharmacological activities such as anti-inflammation, antitumor, and antioxidant properties [7–9]. According to Oboh et al. [10], extract of ginger rhizomes inhibits acetylcholinesterase activity (key regulatory enzyme involved in neurodegeneration)* in vitro*. Furthermore, volatile oil of ginger has been reported to influence both cell-mediated immune response and nonspecific proliferation of T lymphocyte [11].

Turmeric (*Curcuma longa* Linn) is one of the main spices belonging to the family of Zingiberaceae, used as medicine, condiment, and cosmetic worldwide and valued as a functional food because of its health promoting potentials [12]. It is comprised of a group of three curcuminoids: curcumin (diferuloylmethane), demethoxycurcumin, and bisdemethoxycurcumin, as well as volatile oils (tumerone, atlantone, and zingiberone), sugars, proteins, and resins. Several studies have shown that essential oil from turmeric has significant biological activities including antifungal, insect repellent, antibacterial, antimutagenic, anticarcinogenic, antioxidant, anti-inflammatory, and antinociceptive properties [13–15].

More recently, there has been growing attention on natural anti-inflammatory agents capable of reducing or preventing neuroinflammation, coupled with the fact that there are few pharmacological studies regarding the use of essential oils from ginger and turmeric rhizomes in the management/prevention of neurodegenerative diseases. Hence, the anti-inflammatory effect of essential oil from ginger and turmeric rhizomes in cadmium induced neurotoxicity in rats was evaluated by assessing their effect on some cytokines and hippocampus and prefrontal cortex acetylcholinesterase (AChE) and adenosine deaminase (ADA) activities (important enzymes relevant in the management/prevention of neurodegenerative diseases).

## 2. Materials and Methods

### 2.1. Chemicals

Cadmium chloride (CdCl_2_) was obtained from Oxford Laboratory, Mumbai, India, and solubilized in normal saline. Acetylthiocholine iodide, adenosine, 5,5′-dithiobis, 2-nitrobenzoic acid (DTNB), and Tris-HCl were purchased from Sigma–Aldrich, St. Louis, MO, USA. All other reagents used in this study were of analytical grade and the water was glass distilled.

### 2.2. Sample Collection and Extraction of Essential Oils

Fresh Nigeria ginger (*Zingiber officinale* Roscoe) and turmeric (*Curcuma longa *Linn) rhizomes were purchased from the local market of Ado-Ekiti, Nigeria. The rhizomes were identified and a voucher specimen was deposited with the Herbarium at the Department of Plant science laboratory, Faculty of science, Ekiti State University, Ado-Ekiti, Nigeria.

Thereafter, fresh ginger and turmeric rhizomes (100 g of each) were subjected to hydrodistillation in a Clevenger apparatus for 3 h. The obtained oils were dried over anhydrous sodium sulfate. The oils were stored at 4°C in a refrigerator until used for experiment.

### 2.3. Animals

Adult male albino rats (twelve weeks old) were obtained from the animal breeding unit at College of Medicine, Afe Babalola University, Nigeria. The rats were kept under good ventilation and illumination condition and received standard diet and water* ad libitum*. Animals were cared for according to US National Institute of Health (NIH) ethical guidelines. Treatment protocol was in accordance with the ethical requirement of the Animal Use and Care Committee of Afe Babalola University, Ado-Ekiti, Nigeria.

### 2.4. Experimental Design

In the present study, the Cd was administered according to Goncalves et al. [16] while the choice of the essential oil doses (50 mg/kg) was made based on a preliminary acute toxicity study where we obtained beneficial results of this compound in brain of rats (Data not shown). After acclimatization period, sixty (60) rats were divided randomly into six groups of ten (10) animals each; namely,

- Group I serves as the control and rats of this group received daily administration of 0.3% DMSO (vehicle of essential oil) orally plus saline (1 mL/kg) via intraperitoneally (i.p.).
- Group II received daily injections of saline (1 mL/kg) i.p. plus essential oil from turmeric (50 mg/kg) orally.
- Group III received daily injections of saline (1 mL/kg) i.p. plus essential oil from ginger (50 mg/kg) orally.
- Group IV, received daily administration of 0.3% DMSO (vehicle of essential oil) orally plus Cd (2.5 mg/kg) i.p.
- Group V received daily administration of essential oil from turmeric (50 mg/kg) orally plus Cd (2.5 mg/kg) via i.p.
- Group VI received daily administration of essential oil from ginger (50 mg/kg) orally plus Cd (2.5 mg/kg) via i.p.

The experiment lasted for 14 days; both Cd and essential oil were administered daily. Essential oil was prepared in DMSO (0.3%) and administered orally 30 min before Cd injection. After the last day of treatment (14^th^ day), animals were fasted overnight and euthanized according to the approved protocol. The whole brains as well as prefrontal cortex and hippocampus were isolated and homogenized in 100 mM potassium phosphate, pH 7.5. The homogenates were centrifuged at 14,000 × g for 10 min at 4°C, and the supernatant was used for the subsequent enzymatic assays.

### 2.5. Quantification of Cytokine Levels Using ELISA—Marker for Inflammation

Whole brain tissue cytokine (TNF-*α*, IL-6, and IL-10) levels were determined by ELISA using Quantikine Immunoassay kits (R&D systems) according to the manufacturer's instructions. Briefly, 96-well microplates were sensitized with primary antibody at room temperature for 30 min. Then, the tissue sample was added and incubated for another 30 min and wash. After washing, secondary antibody conjugated with peroxidase was added and incubated. Thereafter, the cytokine concentration was measured using an ELISA plate reader.

### 2.6. Biomarkers for Neurotoxicity

#### 2.6.1. Enzymatic Assay of Acetylcholinesterase (AChE) Activity

AChE activity was measured using a 96-well microplate reader based on Ellman et al. [17]. In the 96-well plates, 25 *µ*L of tissue homogenate, followed by 125 *µ*L of 3 mM DTNB and 25 *µ*L of buffer, was added. The resultant mixture was incubated at 30°C for 15 min. Finally, 25 *µ*L of 15 mM substrate solution was added and the absorbance was measured at 405 nm every 30 s for 2 mins using a Microplate Spectrophotometer (Molecular Devices, Sunnyvale, CA). Absorbance was plotted against time and the enzyme activity was calculated.

#### 2.6.2. Adenosine Deaminase (ADA) Activity Assay

ADA activity determination was performed as described by Guisti and Galanti [18]. In brief, 50 *μ*L of tissue homogenate, followed by 50 *µ*L of 21 mM of adenosine, pH 6.5, was incubated at 37°C for 60 min. Absorbance was measured at 630 nm using a Microplate Spectrophotometer (Molecular Devices, Sunnyvale, CA). Enzyme activity was calculated in units per liter (U/L).

### 2.7. Gas Chromatography-Mass Spectrometry (GC/MS) Analysis

The samples were analyzed for volatile compounds using 7820A gas chromatograph coupled to 5975C inert mass spectrometer (with triple axis detector) with electron-impact source (Agilent Technologies). The stationary phase of separation of the compounds was HP-5 capillary column coated with 5% Phenyl Methyl Siloxane (30 m length x 0.32 mm diameter x 0.25 *µ*m film thickness) (Agilent Technologies). The carrier gas was Helium (He) used at constant flow rate of 1.6 mL/min at an initial nominal pressure of 2.84 psi and average velocity of 46 cm/sec. 1 *µ*L of the samples was injected in split less mode at an injection temperature of 260°C. Purge flow was 21.5 mL/min at 0.50 min with a total flow of 25.8 mL/min; gas saver mode was switched on. Oven was initially programmed at 60°C (1 min) and then ramped at 4°C/min to 110°C (3 min), then 8°C/min to 260°C (5 min), and 10°C/min to 300°C (12 min). Run time was 56.25 min with a 3 min solvent delay. The mass spectrometer was operated in electron-impact ionization mode at 70 eV with ion source temperature of 230°C, quadrupole temperature of 150°C, and transfer line temperature of 280°C.

The identification of the compounds was achieved on the basis of retention time, Kovats Index, literature reported retention index using a homologous series of n-alkanes (C8–C25 hydrocarbons, Polyscience Corp., Niles, IL), coinjection with standards (Sigma Aldrich, St. Louis, MO), and mass spectra library search (NIST, Wiley, and Nbs) and by comparing with the mass spectral literature data.

### 2.8. Total Protein Concentration

Protein concentration was determined according to the method of Bradford (1976) using albumin as standard. This was done to normalize the results obtained.

### 2.9. Statistical Analysis

All the results were represented as mean ± SEM performed in triplicate. Statistical analysis used was software of GraphPad Prism 4.0 (GraphPad Software, Inc., San Diego, CA). One-way analysis of variance (ANOVA), followed by Duncan's multiple range tests, was used and probability of P < 0.05 was considered to be statistically significant.

## 3. Results

### 3.1. Cytokine Biomarkers

**Figure 1:**
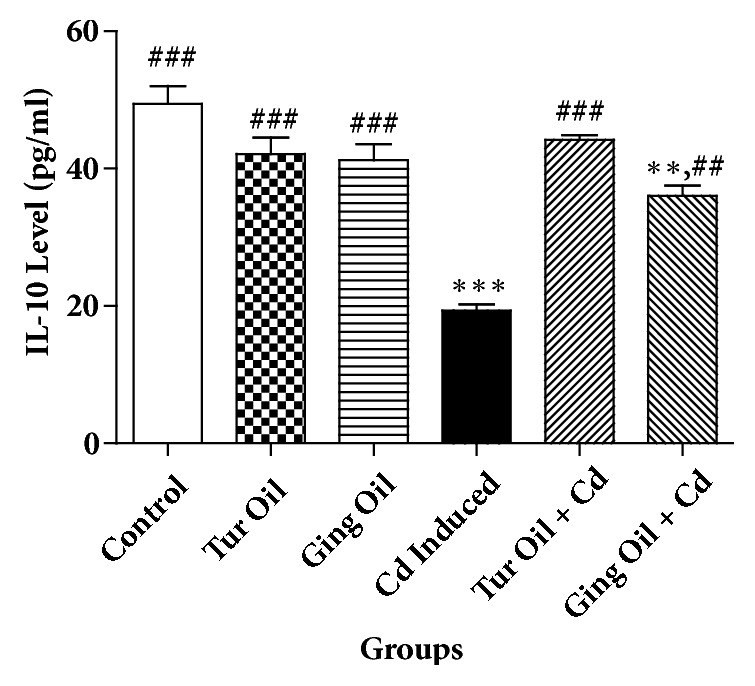
Effect of essential oil from ginger (*Zingiber officinale*) and turmeric (*Curcuma longa*) on interleukin-10 (IL-10) level in cadmium-induced neuroinflammation in rats. Data represent the mean ± SEM of a group of eight rats (^*∗∗*^P < 0.01, ^*∗∗∗*^P < 0.001 vs control; ^##^P < 0.01, ^###^P < 0.001 vs Cd Induced).** Key**: Control: received normal saline + vehicle. Tur Oil: received normal saline + turmeric essential oil (50 mg/kg). Ging Oil: received normal saline + ginger essential oil (50 mg/kg). Cd Induced: received Cd + vehicle. Tur Oil + Cd: received Cd + turmeric essential oil (50 mg/kg). Ging Oil + Cd: received Cd + ginger essential oil (50 mg/kg).

**Figure 2:**
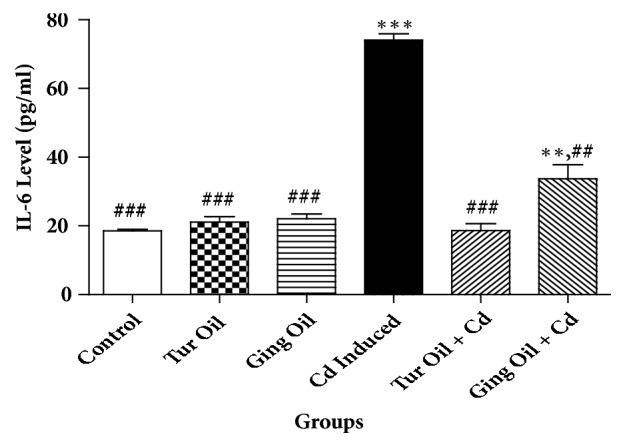
Effect of essential oil from ginger (*Zingiber officinale*) and turmeric (*Curcuma longa*) on interleukin-6 (IL-6) level in cadmium-induced neuroinflammation in rats. Data represent the mean ± SEM of a group of eight rats (^*∗∗*^P < 0.01, ^*∗∗∗*^P < 0.001 vs control; ^##^P < 0.01, ^###^P < 0.001 vs Cd Induced).** Key**: Control: received normal saline + vehicle. Tur Oil: received normal saline + turmeric essential oil (50 mg/kg). Ging Oil: received normal saline + ginger essential oil (50 mg/kg). Cd Induced: received Cd + vehicle. Tur Oil + Cd: received Cd + turmeric essential oil (50 mg/kg). Ging Oil + Cd: received Cd + ginger essential oil (50 mg/kg).

**Figure 3:**
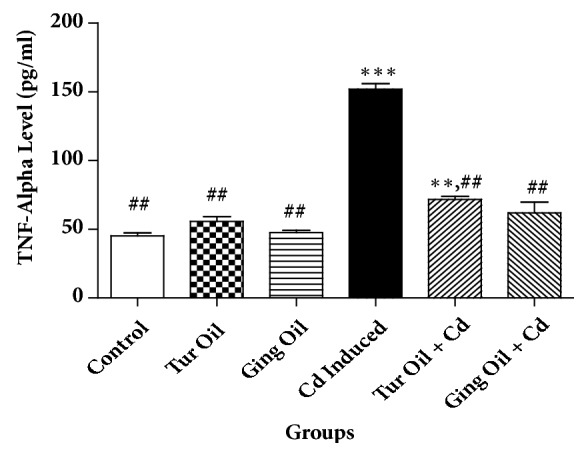
Effect of essential oil from ginger (*Zingiber officinale*) and turmeric (*Curcuma longa*) on Tumor necrosis factor alpha (TNF-*α*) level in cadmium-induced neuroinflammation in rats. Data represent the mean ± SEM of a group of eight rats (^*∗∗*^P < 0.01, ^*∗∗∗*^P < 0.001 vs Control; ^##^P < 0.01vs Cd Induced).** Key**: Control: received normal saline + vehicle. Tur Oil: received normal saline + turmeric essential oil (50 mg/kg). Ging Oil: received normal saline + ginger essential oil (50 mg/kg). Cd Induced: received Cd + vehicle. Tur Oil + Cd: received Cd + turmeric essential oil (50 mg/kg). Ging Oil + Cd: received Cd + ginger essential oil (50 mg/kg).

The effects of essential oil from Nigeria ginger and turmeric rhizomes on some cytokines [interleukin 1 (IL-10), interleukin 6 (IL-6), and tumor necrosis factor alpha (TNF-*α*)] level in cadmium treated rats are shown in Figures 1–3. Cadmium administration caused a significant (P < 0.05) reduction in IL-10 level with a concomitant increase in IL-6 and TNF-*α* level when compared to control. However, cotreatment with essential oil from ginger and turmeric rhizome alters the level of these cytokines by preventing a decrease in IL-10 and causing a reduction in IL-6 and TNF-*α* levels when compared with Cd untreated group (Figures 1–3).

### 3.2. Acetylcholinesterase Activity

**Figure 4:**
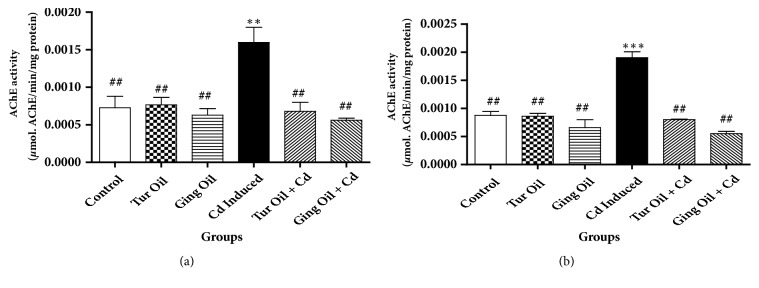
Effect of essential oil from ginger (*Zingiber officinale*) and turmeric (*Curcuma longa*) on prefrontal-cortex (a) and hippocampus (b) acetylcholinesterase (AChE) activity in cadmium-induced neuroinflammation in rats. Data represent the mean ± SEM of a group of eight rats (^*∗∗*^P < 0.01, ^*∗∗∗*^P < 0.001 vs control; ^##^P < 0.01vs Cd Induced).** Key**: Control: received normal saline + vehicle. Tur Oil: received normal saline + turmeric essential oil (50 mg/kg). Ging Oil: received normal saline + ginger essential oil (50 mg/kg). Cd Induced: received Cd + vehicle. Tur Oil + Cd: received Cd + turmeric essential oil (50 mg/kg). Ging Oil + Cd: received Cd + ginger essential oil (50 mg/kg).

Figure 4 represent the effect of essential oil from Nigeria ginger and turmeric rhizomes on hippocampus and pre-frontal cortex AChE activity in cadmium treated rats (Figures 4(a) and 4(b)). We observed a significant (P < 0.05) inhibitory effect on both hippocampus and prefrontal cortex AChE activity by the essential oils when compared with Cd exposed group.

### 3.3. Adenosine Deaminase Activity

**Figure 5:**
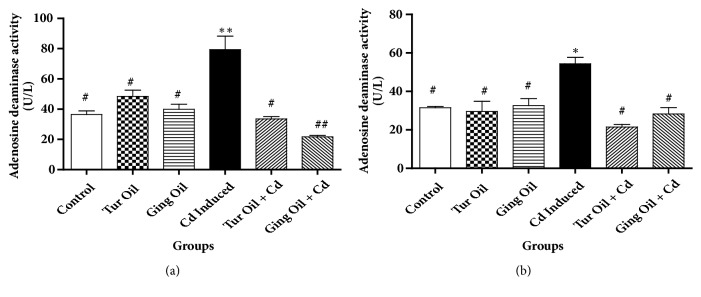
Effect of essential oil from ginger (*Zingiber officinale*) and turmeric (*Curcuma longa*) on prefrontal-cortex (a) and hippocampus (b) adenosine deaminase (ADA) activity in cadmium-induced neuroinflammation in rats. Data represent the mean ± SEM of a group of eight rats (^*∗∗*^P < 0.01, ^*∗∗∗*^P < 0.001 vs control; ^#^P < 0.05, ^##^P < 0.01vs Cd Induced).** Key**: Control: received normal saline + vehicle. Tur Oil: received normal saline + turmeric essential oil (50 mg/kg). Ging Oil: received normal saline + ginger essential oil (50 mg/kg). Cd Induced: received Cd + vehicle. Tur Oil + Cd: received Cd + turmeric essential oil (50 mg/kg). Ging Oil + Cd: received Cd + ginger essential oil (50 mg/kg).

Furthermore, cadmium administration caused a significant (P < 0.05) increase in hippocampus and prefrontal cortex ADA activity when compared to control. However, treatment with essential oil from ginger and turmeric rhizome, respectively, prevents an increase in ADA activity when compared with Cd untreated group (Figures 5(a) and 5(b)).

### 3.4. GC-MS Analysis

In order to justify the efficacy of the essential oils, we characterized the oils for volatile oil components using gas chromatography analysis according to their retention indices (RI) on a HP-5MS column and the observed results are listed in Table 1. The result revealed that the major essential oils from turmeric rhizomes are Eucalyptol (76.46%), *α*-Terpinene (4.41%), *γ*-Terpinene (3.32%), p-Cymene (1.31%), and *α*-Terpineol (0.62%) while *α*-Zingiberene (17.43%), *β*-Sesquiphellandrene (3.10%), Eucalyptol (2.75%), Furfaral (1.76%), *α*-Terpineol (1.35%), endo-Borneol (1.31%), Limonene (1.21%), Thunbergol (0.84%), Citral (0.56%), Oxirane (0.45%), Caryophyllene oxide (0.42%), Nerolidol (0.31%), exo-Norborneol (0.27%), cis-Verbenol (0.12%), Linaloloxide (0.06%), and Squalene (0.02%) are the major essential oil compounds detected in ginger rhizomes.

## 4. Discussion

The production of amyloid beta proteins by *β*-secretase (BACE) and *γ*-secretase in frontal cortex and hippocampus has been linked to the pathogenesis of AD [2]. Several studies have established the relationship between neuroinflammation and AD linking the involvement of heavy metals [2, 3, 19, 20].

In the present study, Cd administration caused a reduction in IL-10 (anti-inflammatory cytokine) with a concomitant increase in IL-6 and TNF-*α* (pro-inflammatory cytokine) levels when compared to control. Previous studies have shown that Cd can induced neuroinflammatory pathology by mechanism involving blood-brain barrier leakage, microglia activation and infiltration of immune cells into the brain [2, 19, 20]. It is interesting to note that cotreatment with essential oil from ginger and turmeric rhizomes prevents alteration in the level of these cytokines (Figures 1–3). This is an indication that the essential oils exert immunomodulatory activity and offer neuroprotection against inflammatory processes associated with neurodegeneration. This activity could be attributed to the volatile compounds present (Table 1) that have been extensively studied for their anti-inflammatory properties and their ability to inhibit inflammatory mediators [9, 14, 21, 22].

It is well established that the brain interacts dynamically with the immune system to modulate inflammation through a neural pathway otherwise called cholinergic anti-inflammatory pathway [23]. This pathway consists of the vagus nerve, acetylcholine (ACh) and the *α*7 subunit of the nicotinic acetylcholine receptor [23]. Studies have demonstrated that ACh, a neurotransmitter, attenuates the production of TNF, IL-6, and IL-18 by human macrophages at the posttranscriptional stage and alter IL-10 release, which indicates a direct inhibitory effect of ACh on proinflammatory cytokine production [23–26].

Acetylcholine (ACh) is rapidly hydrolyzed by acetylcholinesterase (AChE) in neural synapses [26]. Considering the anti-inflammatory effect of ACh, it is possible to suggest that AChE activity is an intrinsic regulator of inflammation. In the present study, treatment with essential oils from both ginger and turmeric rhizomes inhibited hippocampus and pre-frontal cortex AChE activity when compared with Cd exposed group (Figure 4). This result highlights the pivotal role of acetylcholine in inflammation and provides efficacy of the essential oil from ginger and turmeric as acetylcholinesterase inhibitor for the treatment of AD associated with inflammatory response. Previous studies have shown that peritoneal injection of acetylcholinesterase inhibitors reduces serum proinflammatory cytokine levels in brain and blood and decreases serum acetylcholinesterase activity in mice [27, 28].

It is interesting to note that both acetylcholine (ACh) and adenosine triphosphate (ATP) are co-released from the same motor nerve endings in the CNS and have been recognised as an important neurotransmitters involved in the process of neuroimmunomodulation [29–31]. ATP acts as a fast excitatory neurotransmitter and presynaptic neuromodulator [32]. Its breakdown product, adenosine, plays an important modulatory role in neuronal activity and neuroprotective actions in pathological conditions [32, 33]. In the present study, adenosine deaminase (ADA) activity (an important enzyme involves in the regulation of adenosine level) was increased in hippocampus and pre-frontal cortex of Cd-treated rats when compared to control. However, treatment with essential oil from ginger and turmeric rhizome respectively prevents an increase in ADA activity when compared with Cd treated group (Figure 5). This action may lead to an increased extracellular adenosine level and offers neuroprotection during inflammatory response.

In conclusion, this study documents that treatment with essential oil from ginger and turmeric rhizomes exert immunomodulatory effect in Cd exposed rat. The inhibition of AChE and ADA activities by these oils could suggest possible mechanism of action for their anti-inflammatory properties. Hence, essential oil from ginger and turmeric rhizomes could be harnessed as anti-inflammatory supplements for the management/prevention of neurodegenerative diseases associated with inflammation.

## Figures and Tables

**Table 1:** Chemical composition found in the essential oils from Nigeria ginger and turmeric rhizomes.

Compound Name	Ginger		Turmeric	
	Retention time (min)	Percent total of compound (%)	Retention time (min)	Percent total of compound (%)
exo-Norborneol	3.663	0.27	–	–
Eucalyptol	5.194	2.75	5.200	76.46
Linaloloxide	6.511	0.06	–	–
endo-Borneol	8.589	1.31	–	–
Furfural	9.135	1.76	–	–
*α*-Terpineol	9.343	1.35	9.331	0.62
Limonene	12.625	1.21	–	–
p-Cymene	–	–	13.061	1.31
*γ*-Terpinene	–	–	14.350	3.32
Oxirane	18.358	0.45	–	–
Citral	18.869	0.56	–	–
*α*-Terpinene	–	–	19.151	4.41
cis-Verbenol	23.368	0.12	–	–
Thunbergol	23.961	0.84	–	–
Squalene	25.220	0.02	–	–
Caryophyllene oxide	25.504	0.42	–	–
Nerolidol	28.205	0.31	–	–
*α*-Zingiberene	43.08	17.4	–	–
*β*-Sesquiphellandrene	44.85	3.10	–	–
Other components	–	100%	–	100%

## Data Availability

The raw/processed data required to reproduce these findings are available but cannot be shared at this time.
